# Winter warming delays dormancy release, advances budburst, alters carbohydrate metabolism and reduces yield in a temperate shrub

**DOI:** 10.1093/aobpla/plv024

**Published:** 2015-03-23

**Authors:** Majken Pagter, Uffe Brandt Andersen, Lillie Andersen

**Affiliations:** 1 Department of Food Science, Aarhus University, Kirstinebjergvej 10, DK-5792 Aarslev, Denmark; 2 Present address: Department of Chemistry and Bioscience, Aalborg University, Fredrik Bajers Vej 7H, DK-9220 Aalborg East, Denmark

**Keywords:** Climate change, freezing tolerance, overwintering, phenology, plant–climate interactions, *Ribes nigrum*, soluble sugars

## Abstract

Global climate models predict an increase in the mean surface air temperature, with a disproportionate increase during winter. Since temperature is a major driver of phenological events in temperate woody perennials, warming is likely to induce changes in a range of these events. We investigated the impact of slightly elevated temperatures (+0.76 °C in the air, +1.35 °C in the soil) during the non-growing season (October–April) on freezing tolerance, carbohydrate metabolism, dormancy release, spring phenology and reproductive output in two blackcurrant (*Ribes nigrum*) cultivars to understand how winter warming modifies phenological traits in a woody perennial known to have a large chilling requirement and to be sensitive to spring frost. Warming delayed dormancy release more in the cultivar ‘Narve Viking’ than in the cultivar ‘Titania’, but advanced budburst and flowering predominantly in ‘Titania’. Since ‘Narve Viking’ has a higher chilling requirement than ‘Titania’, this indicates that, in high-chilling-requiring genotypes, dormancy responses may temper the effect of warming on spring phenology. Winter warming significantly reduced fruit yield the following summer in both cultivars, corroborating the hypothesis that a decline in winter chill may decrease reproductive effort in blackcurrant. Elevated winter temperatures tended to decrease stem freezing tolerance during cold acclimation and deacclimation, but it did not increase the risk of freeze-induced damage mid-winter. Plants at elevated temperature showed decreased levels of sucrose in stems of both cultivars and flower buds of ‘Narve Viking’, which, in buds, was associated with increased concentrations of glucose and fructose. Hence, winter warming influences carbohydrate metabolism, but it remains to be elucidated whether decreased sucrose levels account for any changes in freezing tolerance. Our results demonstrate that even a slight increase in winter temperature may alter phenological traits in blackcurrant, but to various extents depending on genotype-specific differences in chilling requirement.

## Abstract (precis)

Global climate models predict an increase in the mean surface air temperature, with a disproportionate increase during winter. This study documents that even a very modest temperature increase during the colder periods of a plant's annual cycle may delay dormancy release and advance bud burst and flowering in blackcurrant, but the magnitude of the responses varies between genotypes differing in chilling requirement. Winter warming additionally has a large carryover effect into the growing season by reducing fruit yield the following summer.

## Introduction

Global climate models predict an increase in the mean surface air temperature, frequency and severity of erratic temperature events during this century. Within Europe, temperatures are predicted to increase disproportionately during winter (Christensen *et al.* 2007; Gu *et al.* 2008). Since temperature is a major driver of phenological events in temperate woody perennials (Fitter and Fitter 2002; Parmesan 2006), milder weather during the colder periods of plants’ annual cycle is likely to induce changes in a range of these events. Including development of freezing tolerance in autumn (cold acclimation), dormancy, loss of acclimated freezing tolerance in spring (deacclimation), budburst and flowering. Proper timing of phenological events is an essential trait affecting plant mortality, annual growth and reproductive success (Fu *et al.* 2012; Chung *et al.* 2013). Hence, the magnitude of winter climate change effects on phenological traits may have broad implications for the structure and functioning of forest and landscape ecosystems and the sustainability of horticultural production systems.

In autumn, temperate perennials cold acclimate, whereby they become increasingly tolerant to subzero temperatures. Maximum freezing tolerance is reached mid-winter, while with increasing temperatures in spring plants lose acclimated freezing tolerance by deacclimation (Weiser 1970). Cold acclimation and deacclimation are driven mainly by temperature, therefore climatic warming has been proposed to upset the processes in several ways. First, elevated temperatures may delay or prevent completion of cold acclimation in the autumn and directly accelerate the deacclimation process (Repo *et al.* 1996; Ögren *et al.* 1997; Taulavuori *et al.* 2004). The degree to which warming will influence plant freezing tolerance remains unclear, and may vary between species and ecotypes. For example in *Pinus contorta* Dougl. var *latifolia* Engelm., a +6 °C temperature elevation decreased cold hardiness significantly, whereas it had no effect on *Picea abies* (L.) Karst and *Pinus sylvestris* L. (Ögren 2001). Second, warm temperatures during spring can affect freezing tolerance via promoting ontogenetic development and budburst. This development is irreversible and, once begun, the tissues can no longer increase their hardiness in response to low temperatures (Saxe *et al.* 2001; Rapacz 2002). Indeed, both experimental studies and field recordings have documented an advancement of the growing season in temperate climate zones in response to climate warming (Cook *et al.* 2012; Fu *et al.* 2014; Polgar *et al.* 2014; Vitasse *et al.* 2014). Early onset of spring phenology can increase the risk of tissue damage by subsequent frosts, the likelihood of which is typically high during the early spring (Saxe *et al.* 2001; Jönsson and Bärring 2011).

Concomitant with cold acclimation temperate woody perennials develop dormancy. Dormancy is defined as the inability to initiate growth from meristems (and other organs and cells with the capacity to resume growth) under favourable conditions. Growth is possible only after plants have been exposed to a sufficient amount of chilling temperatures (Rohde and Bhalerao 2007). Some experimental studies suggest that warm temperatures during the dormancy period (typically fall and winter) can delay dormancy release, thereby delaying spring events such as budburst and flowering (Luedeling *et al.* 2011; Fu *et al.* 2012). Hence, warming is expected to have contrasting effects on dormancy release and spring phenology, suggesting that the effect of warming on dormancy release could temper spring phenological advances driven by climate warming.

Cold acclimation is a complex process, which involves extensive modification of the plant metabolome. Carbohydrate metabolism is a particularly prominent component of the reprogramming of the metabolome at low temperatures (Guy *et al.* 2008), and changes in the content of soluble carbohydrates are mechanistically linked to transitions in plant freezing tolerance (Kasuga *et al.* 2007; Poirier *et al.* 2010). Mobilization of starch reserves into soluble carbohydrates during cold acclimation has extensively been documented in a variety of plants species. In contrast, during spring, starch is re-synthesized and mobilized for renewed growth, providing energy and building blocks before significant photosynthesis occur (Cheng and Fuchigami 2002; Charrier and Améglio 2011). Hence, the maintenance of freezing tolerance may compete with the active growth in spring for carbohydrates. Hitherto the primary explanation for reduced mid-winter freezing tolerance and accelerated deacclimation at elevated temperatures is the consumption of soluble sugars due to increasing respiration rates (Ögren 1996; Taulavuori *et al.* 1997).

Blackcurrant (*Ribes nigrum* L.) is an important soft fruit crop of cold and temperate regions. Initiation of floral primordia in blackcurrant is formed in late summer/early autumn shortly after extension growth has slowed down (Tinklin *et al.* 1970). The flower initials then develop to anthesis during the following spring. Blackcurrant flowers in early spring and spring frost can kill flowers (Dale 1987). This has led to the introduction of frost-tolerant late-flowering cultivars with increased chilling requirements (Atkinson *et al.* 2013), which nowadays is causing increasing concern in some parts of Europe that the performance of blackcurrant may be adversely affected by mild winters with insufficient winter chill (Hedley *et al.* 2010). Hence, in blackcurrant warming might not result in a straightforward advancement of budburst because of the contrasting effects of warming on the accumulation of chill units and spring phenology, making it an interesting model crop in relation to climate change aspects in temperate regions.

The aim of the present study was to examine the consequences of slightly elevated temperatures during the winter season on a range of phenological traits in blackcurrant, and to assess to which extent the effects of winter warming varies between two genotypes, which are expected to differ in their chilling requirement. It was hypothesized that (i) winter warming advances spring phenology, but the magnitude varies between genotypes depending on their chilling requirement and hence the impact of warming on dormancy release, and (ii) elevated winter temperatures decreases mid-winter freezing tolerance and accelerates deacclimation, which is reflected by decreasing amounts of soluble carbohydrates associated with freezing tolerance.

## Methods

### Experimental set-up and plant material

The experimental set-up was established in autumn 2012 at the Department of Food Science, Aarhus University in Aarslev, Denmark (latitude 55°18′N), consisting of two plots representing a control plot (ambient temperature) and a warming plot (elevated temperature). The two plots were situated next to each other and were identical with respect to size and layout. The warming plot was surrounded by a *∼*1.3-m tall wind shelter consisting of green polyethylene net. Warming was conducted for *∼*6 months (late October 2012–mid-April 2013) with 240 m of temperature-controlled heating cable (producing maximum 83 W m^−2^). The cable was laid out on the ground between the plants forming 14 continuous loops. The air temperature in both plots was monitored by PT100 temperature sensors covered by radiation shields at 20, 50 and 80 cm above the soil surface. Temperature means over 5-min intervals were logged throughout the treatment period. Whenever the temperature at 20 cm in the warming plot was more than 2 °C higher than the corresponding temperature in the control plot, the heating cable was switched off until the temperature difference between the two plots fell below 2 °C. Initially the soil temperature was monitored with Tinytalk temperature loggers (Gemini Data Loggers, Chichester, UK) buried at 15–20 cm depth. In mid-December, the loggers were replaced by temperature sensors similar to the sensors used to measure the air temperature, as some of the loggers were destroyed when harvesting plants. The sensors were pushed ∼15 cm into the soil. The warming treatment started in late October; however, since the temperature at 20 cm initially differed only slightly between the plots, plants in the warming plot were covered by two low tunnels of transparent polyethylene net fixed on an iron frame on 26 November. The two tunnels were placed next to each other. The net has a shading effect of 15 %; however, upon establishment of the tunnels, plants had shed their leaves and were presumably unable to detect the light level. The warming treatment allowed the plants to be exposed to the natural rainfall, whereas the covering partly prevented snow entering the warming plot. During the periods of snowfall, the cover of the warming plot was checked once a day and any snow that remained on it was removed. The low tunnels covering the plants in the warming plot were removed on 17 April, to avoid any shading effects during the growing season. At this time the buds had green tips. At the same time, the warming treatment was terminated by switching off the heating cable. In addition to the temperature recordings in the plots, local air temperature data were obtained from the Department's climate station, which is operated by the Danish Meteorological Institute.

The experiment was carried out using 3-year-old vegetatively propagated and commercially produced *R. nigrum* cv. Narve Viking and Titania plants. Plants were produced from cuttings, which were put in the field in May and left until winter, where they were dug up and moved to a cold store. The following spring, the cuttings were transplanted into 3.5 L pots containing sphagnum peat, in which they were grown thereafter. ‘Titania’ is a Swedish cultivar (Kikas *et al.* 2011). To our knowledge, the chilling requirement of ‘Titania’ has not been determined, but we have previously observed that field-grown ‘Titania’ is released from dormancy in early December (L. Andersen, unpublished), indicating a modest chilling requirement. In contrast, ‘Narve Viking, which originates from Norway, is known to be a high-chilling-requiring genotype (Sønsteby and Heide 2011). Before start of the experiment plants were grown outside. Hence, they initiated cold acclimation under natural conditions. Before initiation of the warming treatment ∼84 plants of each cultivar were placed in each of the experimental plots with pots buried in the soil to avoid root frost injuries. Each cultivar was placed in two diagonal groupings in each plot.

### Freezing tolerance of stems and buds

In October, before start of the warming treatment, sampling of shoots for determination of freezing tolerance was carried out once and twice in ‘Titania’ and ‘Narve Viking’, respectively, because slightly less plants of ‘Narve Viking’ than ‘Titania’ were available. After start of the warming treatment freezing tolerance was determined approximately once every month from mid-November 2012 to late May 2013. At each sampling time samples were randomly collected from six plants per cultivar and treatment. Freezing tolerance of stems was determined on one control (4 °C) and seven sub-freezing temperatures using the electrolyte leakage method as described in detail in Pagter *et al*. (2011*b*). In short, rinsed samples were placed in 70 mL test tubes containing 100 µL of demineralized water and incubated in a temperature-controlled freezer. The samples were cooled at a rate of maximum 5 °C per hour to 0 °C and subsequently at 2 °C per hour until the selected temperature was reached. The selected temperature was maintained for 2 h, thereafter samples were withdrawn and thawed overnight on ice at 4 °C. Ions were extracted with demineralized water and the electrical conductivity measured before (EC_frozen_) and after (EC_autoclave_) autoclaving. Relative electrolyte leakage (REL) was calculated as REL = (EC_frozen_ − EC_water_) × 100/(EC_autoclave_ − EC_water_), where EC_water_ is the electrical conductivity of demineralized water.

To determine floral bud freezing tolerance stems with attached axillary buds were pruned to equal lengths (1–2 cm), wrapped in moist paper towels to ensure ice nucleation and inserted into small sealed plastic bags. Bud samples were incubated together with the stem samples in a pre-cooled temperature-controlled freezer on top of an aluminium grating and were subjected to the same freezing profile as stem samples. Two sets of bud samples were prepared and after freezing and thawing, overnight on ice at 4 °C, one set of bud samples with attached stem pieces were incubated at 30 °C for 3–4 days before examining bud damage. Buds were then dissected under a dissecting microscope and assessed for injury. Buds were classified as dead (yellow-brown and/or with a soft water-soaked appearance), alive (green and succulent) or injured (some parts yellow-brownish and other parts green). From the second set of bud samples individual buds were excised and incubated in 0.5 % 2,3,5-triphenyltetrazolium chloride (TTC) solution in 0.05 M phosphate buffer at 30 °C for 24 h in darkness. Following incubation in TTC, the colouration of flower primordia was assessed with a dissecting microscope. Active dehydrogenases in mitochondria reduce colourless TTC to red triphenylformazan (Steponkus and Lanphear 1967), hence bright red and red floral primordia were assessed as vital, primordia with a weakly colouration as less vital and colourless or brownish primordia as dead.

### Carbohydrate analysis

Simultaneously with harvest of shoots for determination of freezing tolerance, three to four uppermost positioned axillary buds and two internodal stem pieces from six plants per treatment and cultivar were harvested. Due to a logistical mistake, stem samples were not harvested in September and October before the start of the warming treatment. Samples were frozen in liquid nitrogen and stored at −80 °C.

Soluble carbohydrates were extracted from freeze-dried and finely ground material prior to analysis using high-performance anion exchange chromatography coupled with pulsed amperometric detection (HPAEC-PAD) on a Dionex ICS-300 (Dionex Corp., Sunnyvale, CA, USA) as described by Kjaer *et al.* (2012). Starch was determined in the remaining pellets after extraction of soluble carbohydrates.

### Dormancy status

The dormancy status of the plants was evaluated once a month from late October to mid-February by transferring potted plants into a greenhouse and inducing bud break at 20 °C day/night and a 16-h photoperiod. At each sampling time, six plants per treatment and cultivar were transferred to forcing conditions. Under the forcing conditions budburst was observed three to four times per week, separately for the apical bud and the four top-most lateral buds of a single shoot. Budburst was recorded using a rating of 0–3, where 0 = green tip not visible, 1 = green tip visible, 2 = green leaves and 3 = green leaves and flowers. At the first sampling time, plants were moved to the forcing conditions right before the start of the warming treatment and hence only plants at ambient temperature were evaluated. At this time, single shoots of ‘Narve Viking’ were evaluated for depth of dormancy. The base of each shoot was placed in water. Few millimetres of the base were cut away weekly in order to prevent embolism caused by a possible proliferation of microorganisms, and the water was changed every 3 days.

### Bud break, flowering and cropping performance

Timing of bud break and flowering in spring was recorded in the same way as the evaluation of depth of dormancy from 8 April to mid-May on 12 plants per treatment and cultivar. The number of flowers originating from the top-most four lateral buds of the same plants was recorded on 24 May and cropping performance of the entire plants was evaluated in the end of July by recording the fruit yield.

### Statistical analysis

Freezing tolerance was estimated as LT_50_ values, the temperature representing 50 % REL. For each sampling, data for all six replicates were fitted by regression analysis (PROC NLIN) to the sigmoid function REL = REL_min_ + (REL_max_ − REL_min_)/(1 + exp(*c*(*d*− *T*))), where REL_min_ is the baseline of REL, REL_max_ is the maximum REL, *c* is the slope of the function at the inflection point *d* and *T* is the treatment temperature. The temperature (*d*) at the inflection point was used as LT_50_ (Pagter *et al.* 2011*a*). It was not possible to estimate the LT_50_ values of any cultivar in the two treatments following the determination of REL in mid-February. The reason for this is unknown, but possibly the selected sub-freezing temperatures did not cover the temperature span around the LT_50_ values sufficiently well to allow estimation of the LT_50_ values. Differences between LT_50_ estimates were taken as significant if the 95% confidence intervals did not overlap.

Differences in the average attainment of each of the four bud stages during forcing at different times during the winter were analysed using a three-way analysis of variance (ANOVA, PROC MIXED of SAS, SAS Institute, Cary, NC, USA). The main effects were cultivar, treatment and days of forcing, as well as their interactions. The effects of cultivar, treatment and number of days of evaluation on budburst and flowering in spring in the field were analysed in the same way.

Differences in concentrations of soluble carbohydrates and starch in buds and stems were analysed using three-way ANOVA and Type III sum of squares (PROC GLM). The main effects were cultivar, treatment and seasonal time (ST), as well as their interactions. Heterogeneities of variance within each cultivar and treatment at each time were tested using Bartlett's test. When necessary, data were log-transformed to ensure homogeneity of variance, but for clarity all data are presented as untransformed. Differences between individual means were identified using Tukey's studentized range test at the 5 % significance level. A non-parametric Kruskall–Wallis test was also used to test differences in glucose and sucrose in buds and fructose, raffinose and starch in buds and stems between cultivars, treatments and harvesting dates. This test was done to supplement the result from the ANOVA, as the assumption concerning similar variance was not fulfilled. The ANOVA and the Kruskall–Wallis test gave similar results when assessing the effects of cultivar, treatment and ST on glucose, sucrose and raffinose in buds and fructose in buds and stems, and only the results of the ANOVA are described. For raffinose in stems and starch in buds and stems, the ANOVA indicated that the contents differed significantly between treatments and cultivars, respectively, whereas the Kruskall–Wallis test did not indicate significant cultivar or treatment differences. Hence, only the results of the Kruskall–Wallis tests are shown. The effects of ST and cultivar on the number of flowers and fruit yield was analysed using two-way ANOVA. The correlation coefficients between LT_50_ values and air temperatures or concentrations of soluble carbohydrates, and between air temperatures and concentration of soluble carbohydrates were examined using Pearson's correlation coefficient (PROC CORR).

## Results

### Climate

**Figure 1.**
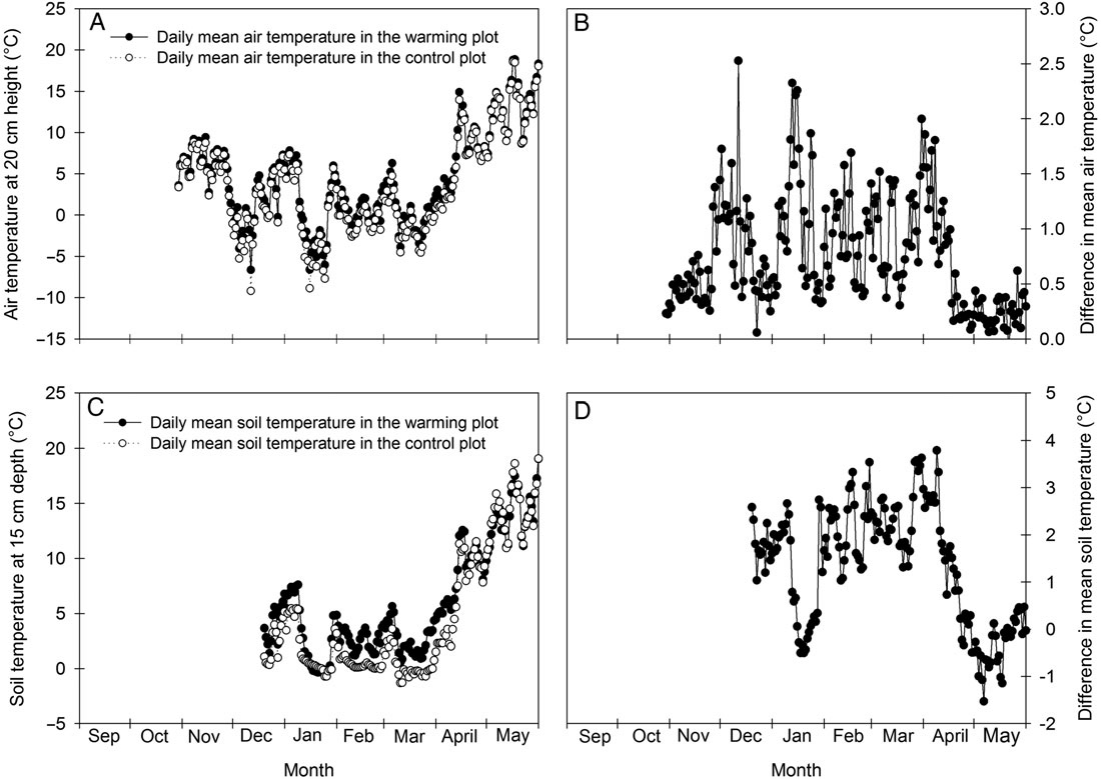
Daily mean air (A) and soil (C) temperatures (°C) at 20 cm height or ∼15 cm depth, respectively, in the control and warming plots during the experimental period. Also shown are the differences in daily mean temperatures between the warming and control plots in the air (B) and the soil (D).

The autumn of 2012 was relatively mild with no freezing events and a daily mean temperature of 6.0 and 6.6 °C in November at 20 cm height in the control and the warming plot, respectively (Fig. 1A). December, January and February had daily mean temperatures just around the freezing point and absolute minimums of −9.2 and −6.7 °C in the control and warming plots, respectively. The spring of 2013 was unusually cold. In March, the daily mean temperature decreased to −0.7 °C in the control plot and 0.3 °C in the warming plot, and 20 and 16 days had average temperatures below zero. In April and May the temperature increased to on average 6.6 and 12.9 °C, respectively, in the two plots, but several days in April and 2 days in May still had temperatures below the freezing point, as evidenced by the daily minimum air temperature recordings (not shown).

The temperatures measured among the plants were slightly higher than the temperature recordings obtained from the climate station (not shown), but the measurements were otherwise in close agreement. The temperature difference at 20 cm height between the control plot and the warming plot was on average 0.76 °C throughout the treatment period (Fig. 1B). It varied between <0.1 and 2.5 °C, being greatest on cold days. From mid-December to the end of the warming treatment, the soil temperature at ∼15 cm depth was on average 1.35 °C higher in the warming plot than in the control plot (Fig. 1C and D). However, for ∼1 week in January, when the soil in the control plot was covered by snow, the soil temperature was lower in the warming than in the control plot. Hence, the experimental set-up with heating cables served the purpose to increase the temperature slightly under outdoor condition.

### Depth of dormancy

**Figure 2.**
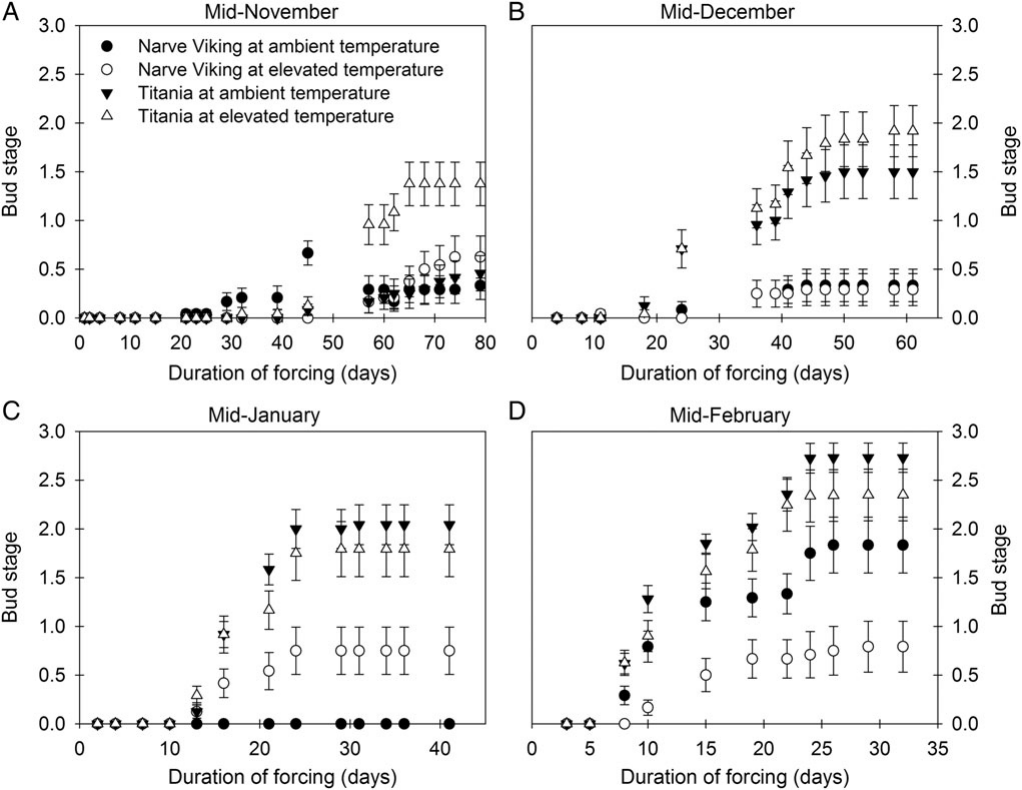
Average attainment of each of the four bud stages of lateral buds of *R. nigrum* ‘Narve Viking’ and ‘Titania’ following varying time of forcing, in a permissive warming environment, at different times during the winter season. Prior to forcing plants were exposed to ambient or slightly elevated temperatures. Values are means ± SE of *n* = 6 shoots.

Right before the start of the warming treatment, in late October, 2 months of forcing did not induce budburst in either cultivar, showing that the plants were endo-dormant at the beginning of the treatment (data not shown). The effect of the warming treatment at different times during the winter season on the average attainment of each of the four bud stages of lateral buds is presented in Fig. 2 and Table 1, when measured after different durations of forcing. In mid-November, both cultivars at ambient temperature and ‘Narve Viking’ at elevated temperature were endo-dormant, with only minor signs of bud development after lasting forcing. However, from 55 days of forcing onwards, buds of ‘Titania’ at elevated temperature developed green tips or leaves (Fig. 2A). In mid-December, buds of ‘Titania’ at both ambient and elevated temperature started to break after ∼20 days of forcing, with no effect of the warming treatment on bud development. Lateral buds of ‘Narve Viking’ in both treatments remained endo-dormant (Fig. 2B). Approximately 1 month later warming significantly advanced lateral bud development in ‘Narve Viking’ after 22 days of forcing, although buds did not develop further than to the ‘green tip visible’—stage at most (Fig. 2C). In mid-February lateral buds of ‘Titania’ at both ambient and elevated temperatures broke fast, whereas buds of ‘Narve Viking’ broke significantly slower (Fig. 2D). In both cultivars, warming significantly delayed bud development, with the effect tending to be greatest in ‘Narve Viking’.

**Table 1.** *F*-values and significance of a three-way ANOVA showing the effects of cultivar (‘Narve Viking’ vs. ‘Titania’), treatment (ambient vs. elevated temperatures), days of forcing and their interactions on the average attainment of each of the four bud stages during forcing of *R. nigrum* at different times during the winter season. *P < 0.05; **P < 0.01; ***P < 0.001; ns, not significant.

Seasonal time	Main factor			Interactions			
	Cultivar (C)	Treatment (T)	Days of forcing (DOF)	C×T	C×DOF	T×DOF	C×D×DOF
November	1.89 ns	2.92 ns	31.51***	2.73 ns	5.36***	8.84***	3.72***
December	24.14***	0.20 ns	26.19***	0.40 ns	12.46***	0.23 ns	0.32 ns
January	22.52***	0.68 ns	64.89***	2.41 ns	29.53***	0.78 ns	3.45***
February	19.43***	3.25 ns	144.68***	1.10 ns	19.98***	3.95***	1.51 ns

Terminal buds were released from dormancy earlier than lateral buds in both cultivars. Hence, mid-December most buds reached stage 2, the final stage for terminal buds, after *∼*1 month of forcing, while in mid-January the same stage was reached after ∼20 days (data not shown). Warming advanced budburst of terminal buds by ∼1 week in mid-December, but had no effect on terminal bud development at the other forcing dates.

### Freezing tolerance

**Figure 3.**
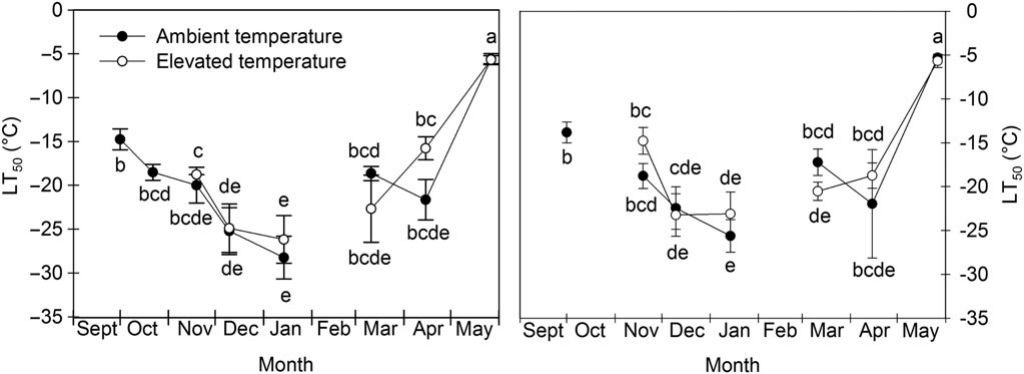
Seasonal changes in cold hardiness estimated as temperatures representing 50 % REL (LT_50_) of stems of *R. nigrum* ‘Titania’ (left) and ‘Narve Viking’ (right) exposed to ambient or slightly elevated winter temperatures. LT_50_ [mean ± SE (°C)] are shown for six plants tested at seven temperatures. Different letters indicate significant differences between treatments and sampling dates for each cultivar separately.

In early October, freezing tolerance of stems was ca. −14 °C in both cultivars and in late October, right before the start of the warming treatment, freezing tolerance of stems of ‘Titania’ was ca. −19 °C, indicating that the plants had developed significant freezing tolerance before the start of the warming treatment (Fig. 3). Between October 1 and mid-January freezing, tolerance increased in successively later sampling dates reaching a maximum of on average −27 and −24 °C in ‘Titania’ and ‘Narve Viking’, respectively. From mid-January to mid-March, freezing tolerance decreased or tended to decrease in both cultivars and treatments, while in April it remained stable in ‘Narve Viking’ and in ‘Titania’ at ambient temperature, but decreased further in ‘Titania’ at elevated temperature. In late May, at the end of the experiment, stem freezing tolerance had decreased to −5 to −6 °C in both cultivars. In both cultivars and treatments, LT_50_ values determined after initiation of the warming treatment correlated well with the average temperatures at 20 cm since the last sampling date (*r* = 0.76 for ‘Titania’ at ambient and elevated temperatures, *r* = 0.77 for ‘Narve Viking’ at ambient temperature and *r* = 0.90* for ‘Narve Viking’ at elevated temperature, *P < 0.05). Lack of significance for most correlations was probably due to the fact that the correlation analyses only encompassed the six sampling dates where LT_50_ values were determined in both treatments. Throughout the warming period stem freezing tolerance tended to be lower in plants at elevated temperature than in plants at ambient temperature. However, differences were not statistically significant.

None of the viability tests were suitable for assessing cold injury of buds. Regardless of collection date, none of the buds examined exhibited visible browning of the floral primordia after exposure to lethal temperatures. Floral primordia generally appeared turgid, but some buds, especially those subjected to the most severe freezing temperatures, exhibited signs of water soaking of tissues. Following incubation in TTC the majority of primordia developed coloured formazan, irrespective of the freezing temperature, making it impossible to distinguish between vital, subvital and dead floral primordia. Previous attempts to quantify floral injury in *R. nigrum* by visible browning (Takeda *et al.* 1993) or TTC (Warmund *et al.* 1991) were also unsuccessful.

### Carbohydrates

**Figure 4.**
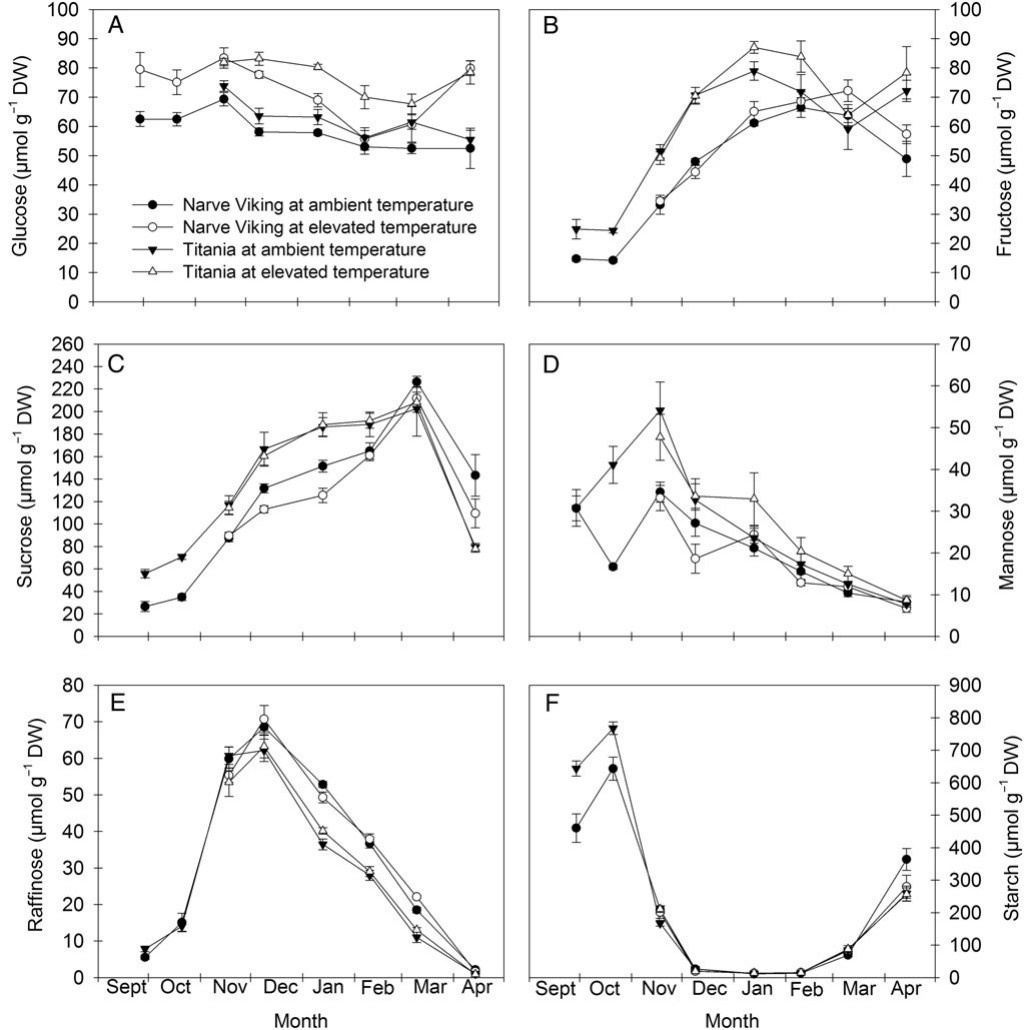
Seasonal changes in concentrations of glucose (A), fructose (B), sucrose (C), mannose (D), raffinose (E) and starch (F) in flower buds of *R. nigrum* ‘Narve Viking’ and ‘Titania’ from the end of September to mid-April the following year. During the winter season, plants were grown at ambient or slightly elevated temperatures. Values are means ± SE of *n* = 5–6.

**Figure 5.**
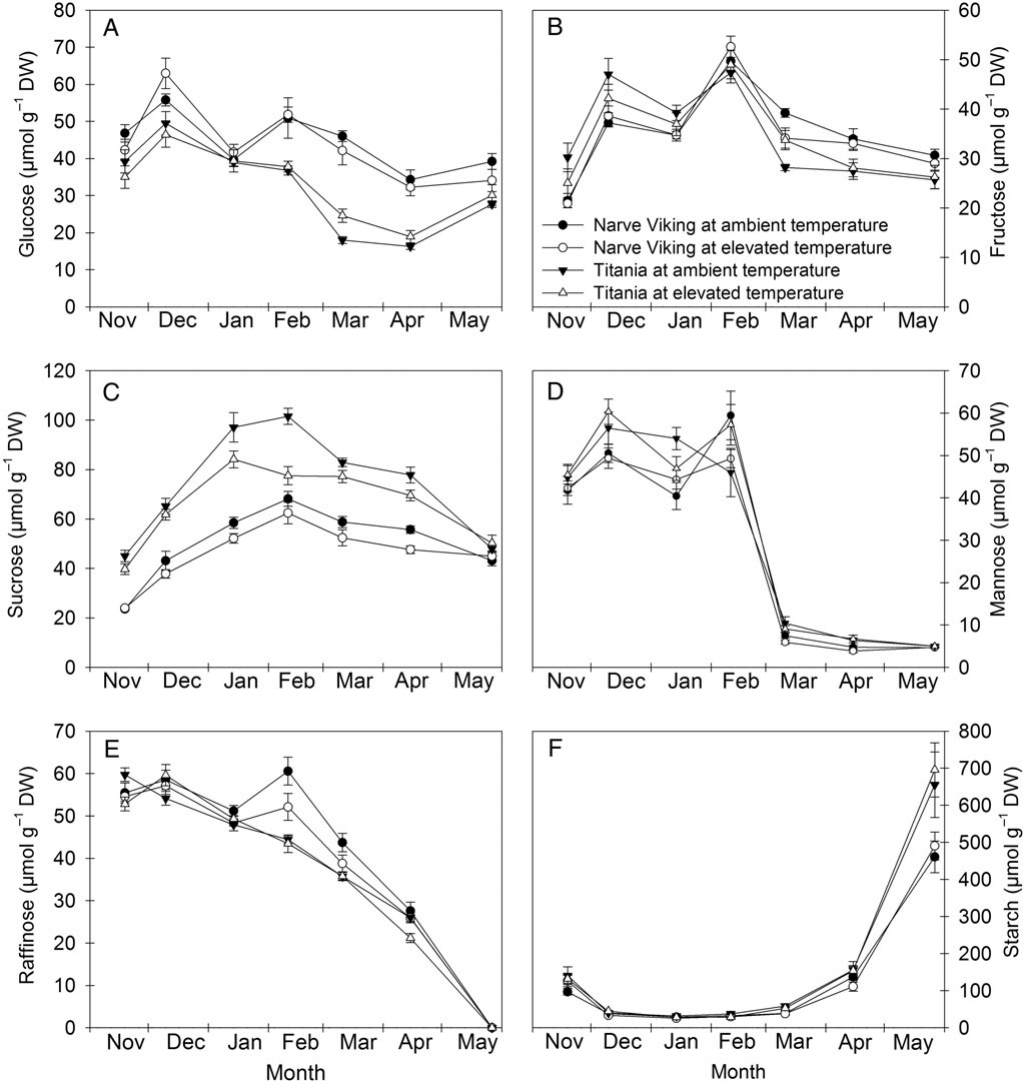
Seasonal changes in concentrations of glucose (A), fructose (B), sucrose (C), mannose (D), raffinose (E) and starch (F) in stems of *R. nigrum* ‘Narve Viking’ and ‘Titania’ from mid-November to the end of May the following year. During the winter season, plants were grown at ambient or slightly elevated temperatures. Values are means ± SE of *n* = 5–6.

The concentrations of starch and five soluble carbohydrates, i.e. glucose, fructose, sucrose, mannose and raffinose, were analysed in buds and stems (Figs 4 and 5, Table 2). Concentrations of carbohydrates varied to different extents depending on the nature of the carbohydrate considered, the organ, the treatment and the cultivar. Sucrose was the primary soluble carbohydrate in both organs and cultivars, displaying a seasonal trend. Its concentration increased during cold acclimation in the autumn, reached a peak in winter (stems) or early spring (buds), and decreased subsequently. Irrespective of treatment and organ the sucrose concentration was significantly higher in ‘Titania’ than in ‘Narve Viking’ at most sampling dates. The concentrations of the hexose sugars were also higher in buds of ‘Titania’ than ‘Narve Viking’ at most sampling dates. In stems, on the other hand, the concentrations of glucose were higher in ‘Narve Viking’ than in ‘Titania’, whereas there was no cultivar difference in the concentrations of fructose. In buds, fructose displayed a seasonal trend, whereas differences in hexose concentrations in stems and glucose in buds did not seem correlated with season or cold hardiness in either cultivar. Warming caused significantly lower sucrose concentrations in stems of both cultivars and buds of ‘Narve Viking’, but increased bud concentrations of glucose and fructose. In stems, the concentration of mannose decreased dramatically in late winter, while in buds the mannose concentration declined more slowly. The concentration of mannose was lower in both buds and stems of ‘Narve Viking’ than that of ‘Titania’, but bud concentrations of mannose were generally lower than the concentrations of the other soluble carbohydrates measured. Contrary to the other soluble carbohydrates measured, concentrations of raffinose were greatest in buds of ‘Narve Viking’, whereas in stems the concentrations did not differ between cultivars. In both cultivars at ambient temperature and ‘Titania’ at elevated temperatures, concentrations of raffinose were highly correlated with stem cold hardiness (*r* = −0.84* in ‘Titania’ at ambient temperature, *r* = −0.89* in ‘Titania’ at elevated temperature and *r* = −0.80* in ‘Narve Viking’ at ambient temperature), whereas in ‘Narve Viking’ at elevated temperature the correlation was not quite significant (*r* = −0.71). Neither the concentrations of mannose nor raffinose in either organ were influenced by warming. Except for mannose, the concentrations of the measured soluble carbohydrates were higher in buds than stems.

**Table 2.** *F*-values and significance of a three-way ANOVA showing the effects of ST (September–April for buds, November–May for stems), treatment (ambient vs. elevated temperatures), cultivar (‘Narve Viking’ vs. ‘Titania’) and their interactions on concentrations of soluble carbohydrates and starch in flower buds and stems of *R. nigrum*. For raffinose in stems and starch in buds and stems are shown *H*-values and significance of Kruskall–Wallis tests. *P < 0.05; **P < 0.01; ***P < 0.001; ns, not significant.

Parameter	Main factors			Interactions			
	Seasonal time (ST)	Treatment (T)	Cultivar (C)	ST×T	ST×C	T×C	ST×T×C
Buds							
Glucose	18.25***	18.17***	122.96***	1.28 ns	4.94***	0.1 ns	1.08 ns
Fructose	33.77***	6.39*	79.15***	1.07 ns	8.3***	0.17 ns	0.46 ns
Sucrose	82.67***	3.95*	12.36***	0.57 ns	15.06***	4.12*	0.52 ns
Mannose	84.39***	0.06 ns	20.28***	1.5 ns	0.92 ns	3.9 ns	0.48 ns
Raffinose	640.41***	0.01 ns	63.52***	2.52*	6.23***	0.04 ns	0.85 ns
Starch	155.77***	0.15 ns	6.94 ns				
Stems							
Glucose	64.14***	0.13 ns	183.9***	1.03 ns	14.88***	2.66 ns	2.31 ns
Fructose	71.72***	0.57 ns	0.13 ns	0.65 ns	8.12***	0.00 ns	2.06 ns
Sucrose	157.6***	25.17***	435.1***	2.14 ns	9.77***	0.52 ns	0.96 ns
Mannose	875.17***	0.8 ns	31.98***	0.98 ns	4.8***	2.4 ns	1.77 ns
Raffinose	140.42***	0.62 ns	2.24 ns				
Starch	146.21***	0.58 ns	3.78 ns

The concentration of starch differed significantly between sampling dates. From initiation of the experiment and until leaf fall (in October) the bud starch concentration increased in both cultivars. Thereafter, concentrations of starch decreased to a winter minimum, which persisted until March. Shortly before budburst and growth of leaves in spring (end of April–early May) starch was re-synthesized in both stems and buds.

### Bud break, flowering and cropping performance

**Figure 6.**
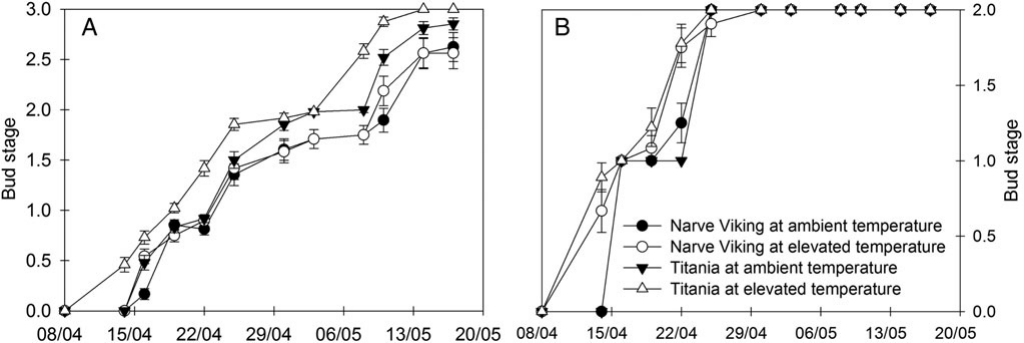
Average attainment of each of the four bud stages of lateral (A) and terminal (B) buds of *R. nigrum* ‘Narve Viking’ and ‘Titania’ during bud break and flowering in the field in spring. During the winter season plants were grown at ambient or slightly elevated temperatures. Values are means ± SE of *n* = 12 shoots.

In spring, lateral buds of ‘Titania’ bursted and flowered earlier than lateral buds of ‘Narve Viking’. In both cultivars, warming significantly advanced leaf unfolding and flowering, but the effect was significantly greater in ‘Titania’ than in ‘Narve viking’ (Fig. 6A, Table 3). For terminal buds, warming advanced budburst by 2 days in both cultivars (Fig. 6B, Table 3).

**Table 3.** *F*-values and significance of a three-way ANOVA on the effects of cultivar (‘Narve Viking’ vs. ‘Titania’), treatment (ambient vs. elevated temperatures), time after start of the recordings and their interactions on the average attainment of each of the four bud stages during leaf unfolding and flowering in spring of lateral and terminal buds of *R. nigrum*. *P < 0.05; **P < 0.01; ***P < 0.001; ns, not significant.

	Main factor			Interactions			
	Cultivar (C)	Treatment (T)	Time (Ti)	C×T	C×Ti	T×Ti	C×T×Ti
Lateral buds	26.95***	6.97*	670.03***	3.06 ns	5.24***	3.21***	2.04*
Terminal buds	0.32 ns	59.70***	525.53***	3.34 ns	1.20 ns	33.58***	1.09 ns

Warming had no effect on the number of flowers from the top-most four lateral buds in either cultivar, but with on average 7.7 flowers per bud ‘Narve Viking’ had significantly more flowers than ‘Titania’, which had on average 5.9 flowers per bud. In both cultivars, warming during the non-growing season caused a significant reduction in fruit yield in the following summer. In ‘Narve Viking’, warming reduced fruit yield (g/plant) by 41 %, while in ‘Titania’ the fruit yield of warmed plants was reduced by 14 % compared with control plants (data not shown). Irrespective of treatment the yield tended to be higher in ‘Narve Viking’ than in ‘Titania’ but differences were not quite significant (*P* = 0.086).

## Discussion

The mean surface air temperature is projected to increase by on average 0.3–0.8 °C by the end of this century (2081–2100) compared with 1986–2005 (IPCC 2013). Hence, the modest increases in the air (0.76 °C) and soil (1.35 °C) temperatures employed in the present study (Fig. 1) are most likely well within the limits of the expected future temperature increase, although the effect of the warming treatment was greatest on cold days, where the importance of a temperature increase presumably is greatest. Despite a modest temperature increase, warming significantly delayed breaking of dormancy in both cultivars, as evidenced by the slower bud development of warmed plants during forcing in February (Fig. 2). This effect was more pronounced in ‘Narve Viking’ than in ‘Titania’. Likely the delaying effect was offset by plenty of additional chilling hours in the second half of February, March and April, as both plants at ambient and elevated temperatures started to burst in April followed by flowering in May in the field (Fig. 6). In ‘Titania’, warming significantly advanced bud development in November after lasting forcing. We hypothesize that this effect of warming on bud development of ‘Titania’ is due to a relatively low chilling requirement, which may partly have been met in November. In accordance, the majority of evaluated buds of ‘Titania’ bursted during forcing in December and January, whereas ‘Narve Viking’ remained dormant.

Several models have been developed for quantifying winter chill, e.g. the Chilling Hours Model, the Utah Model and the Dynamic Model (Luedeling *et al.* 2011). The chill unit accumulation of <7.2 °C chill units model has previously been shown to be the most accurate model in predicting chill satisfaction and timing of bud break of three blackcurrant cultivars in the field (Rose and Cameron 2009). According to this model, which sums up the total number of hours below 7.2 °C, the number of chill units had exceeded 2000 in both plots in mid-February. At this time, ‘Narve Viking’ still did not reach the flowering stage after 1 month of forcing (Fig. 2), reinforcing the perception of this cultivar being a high-chill-requiring genotype (Jones and Brennan 2009; Atkinson *et al.* 2013). However, Jones *et al.* (2013, 2015) recently showed that the effects of varying temperature during chilling and bud break are complex, with warm temperature breaks substantially inhibiting bud development. Hence, the high chilling requirement of ‘Narve Viking’ may partly be a result of the mild autumn and a relatively warm period in late December (Fig. 1). The optimal chilling temperature is generally low in blackcurrant (even below 0 °C in some cultivars) compared with other temperate-zone perennials, but it varies between genotypes (Rose and Cameron 2009; Jones *et al.* 2013), making it difficult to compare chilling requirements across cultivars in a dynamic climate with varying temperatures.

Despite a delay in dormancy release warming significantly advanced leaf unfolding and flowering in both cultivars (Fig. 6), implying that the magnitude of both cultivars' response to warming was greater during the forcing phase in spring than during the dormancy period. The significantly smaller effect of warming on spring phenology of ‘Narve Viking’ compared with ‘Titania’ was likely due to this cultivars’ larger chilling requirement. Hence, by analysing long-term data on phenology and seasonal temperatures from 490 species Cook *et al.* (2012) recently documented that species that do not advance their springtime phenology or delay their timing in response to warming are not less sensitive to spring temperature forcing than species with advanced budburst and flowering. Instead, in these apparently non-responding genotypes dormancy or vernalization responses compensate for spring warming responses that would otherwise advance spring phenology. The chilling requirement of apical buds appeared to be less than that of lateral buds and warming had a smaller effect on the dormancy status and timing of spring bud flush of apical than lateral buds in both cultivars (Fig. 2). This suggests that vegetative and generative buds may differ in their responsiveness to warming both in the dormancy and forcing phases. However, a cautious interpretation of the results of the evaluation of depth of dormancy and bud flush of apical buds is required, as the number of buds evaluated were limited compared with flower buds. In some woody species spring bud flush has been shown to be controlled not only by the air temperature but also by the soil temperature (Greer *et al.* 2006; Hawkins and Dhar 2012), whereas other studies have found no effect of soil temperature on bud phenology (Repo *et al.* 2004; Bailey and Harrington 2006). Hence, it is possible that advanced bud flush and flowering in blackcurrant at elevated temperature was driven by both the increase in air and soil temperature.

Winter warming significantly reduced fruit yield of both cultivars the following summer. This corroborates the hypothesis that a decline in winter chill may cause yield reductions in blackcurrant (Atkinson *et al.* 2013; Jones *et al.* 2013). Interestingly, the yield reduction tended to be greater in ‘Narve Viking’ than in ‘Titania’, suggesting that the decline in yield was indeed associated with the delay in dormancy breaking, and not alterations in the onset of flowering. A recent study has shown that winter warming significantly reduced plant reproductive output for nine temperate indeterminate-growing, multi-inflorescence species, but not for three determinate-growing, single-inflorescence species (Liu *et al.* 2012). The reproductive reduction for the multi-inflorescence species was largely due to a decline in flower number per plant and was attributed to the warming effect on the vernalization. In blackcurrant, however, where flowers are initiated the previous autumn, winter warming had no effect on the number of flowers. This suggests that the yield reduction of warmed plants was due to smaller berries or a decrease in the fruit set ratio. Ghrab *et al.* (2014) have recently shown that warm winter temperatures reduce fruit set of *Prunus persica* in the field. The authors propose that this reduction is due to altered flower bud development and/or inadequate mobilization of stored metabolites.

Slightly elevated winter temperatures did not increase the risk of freeze-induced damage to stems significantly in either cultivar (Fig. 3). In accordance, warming did not alter freezing tolerance of buds of *Betula pendula* (Riikonen *et al.* 2013). In contrast, delayed hardening and/or accelerated dehardening by rising temperature was reported in *P. sylvestris* (Repo *et al.* 1996), *Vaccinium myrtillus* L. (Taulavuori *et al.* 1997) and *Betula pubescens* ssp. *czerepanovii* (Taulavuori *et al.* 2004). Differential tolerance against elevated winter temperatures may be species-specific, but it may also, to some extent, reflect differences in the magnitude of the temperature elevation, with temperature elevations being greater in the studies on *P. sylvestris*, *V. myrtillus* and *B. pubescens* than in the study on *B. pendula* and the present study. Seasonal changes in stem cold hardiness were very similar between cultivars, but absolute stem freezing tolerance tended to be greater in ‘Titania’ than in ‘Narve Viking’.

Plants at elevated temperature showed decreased levels of sucrose in stems of both cultivars and flower buds of ‘Narve Viking’, indicating that even mild winter warming alters plant carbohydrate metabolism (Figs 4 and 5). In buds, the decrease in sucrose was associated with increased concentrations of hexose sugars suggesting that warming induced breakdown of sucrose to glucose and fructose. This is somewhat opposite to observations in *B. pendula*, where two growing seasons of exposure to slightly elevated temperature resulted in decreased bud concentrations of glucose, fructose and sucrose in early December (Riikonen *et al.* 2013). However, carbohydrate changes in response to winter warming may differ considerably between species (Bokhorst *et al.* 2010). In stems, decreased levels of sucrose were not associated with increased levels of hexose sugars, suggesting that warming influences carbohydrate metabolism and/or allocation differently in different organs. Sucrose and raffinose act as cryoprotectants for membranes and proteins during freeze-induced dehydration (Crowe *et al.* 1998; Minorsky 2003), and are considered a determinant factor for cold tolerance and winter survival of plants (Palonen *et al.* 2000; Cox and Stushnoff 2001; Renaut *et al.* 2004). In our study, sucrose displayed a clear seasonal accumulation pattern and although its concentrations were not quantitatively related to stem cold hardiness, it is possible that the small, but insignificant, tendency to reduced hardiness of warmed plants was associated with the loss of cold-protective sucrose. Raffinose, levels of which were significantly inversely related to stem cold hardiness, was not sensitive to elevated temperature. This is in agreement with observations in buds of *B. pendula* (Riikonen *et al.* 2013). However, since biosynthesis of raffinose is largely dependent on the availability of sucrose (Kaplan *et al.* 2007) more severe or longer-lasting warming may potentially affect concentrations of raffinose. In ‘Narve Viking’, the concentration of starch in buds tended to be lower at elevated temperature than at ambient temperature in mid-April shortly before bud break. Although speculative this may suggest that warming can impair starch remobilization in spring.

It has been suggested that in plants grown under generally warmer conditions physiologically acclimation may slightly diminish the effects of greater thermal availability (Eccel *et al.* 2009). In addition, there are studies showing that previously experienced climatic conditions may alter plant performance within the same or in subsequent seasons, indicating the potential importance of preconditions (Kreyling *et al.* 2012; Walter *et al.* 2013). Consequently, we cannot rule out that in a longer-term (years) blackcurrant may respond differently to winter warming than observed in the present study.

## Conclusions

Our study has documented that even mild winter warming modifies phenological traits of blackcurrant, but the magnitude of the responses varies between genotypes differing in chilling requirement. Under the present conditions, the response to warming was greater in spring than during the dormancy phase in both cultivars, but since warming predominantly advanced spring phenology of the relatively low-chilling-requiring genotype ‘Titania’, it is likely that in even warmer winters the response during the dormancy phase will override spring phenological advances in genotypes with large chilling requirements (e.g. ‘Narve Viking’). While it was recently shown that warming may alter reproductive output of herbaceous species, this is the first study to show that a decline in winter chill may cause yield reductions in a woody perennial. The mechanistic reasons underlying the effect of winter warming on reproductive output are unknown, but since it is not attributable to a decline in flower number or altered flowering onset time, it may reflect a change in flower fertility or a physiological response (e.g. metabolic changes) resulting in a reduced fruit set ratio. Contrary to our second hypothesis, warming did not decrease freezing tolerance, although it tended to increase the risk of frost injuries during cold acclimation and deacclimation. Winter warming alters carbohydrate metabolism, but it remains to be elucidated whether decreased sucrose levels account for any small changes in freezing tolerance.

## Sources of Funding

This study was supported by Interreg IVB North Sea Region Programme (2007–2013) through Project ID: 35-2-05-09 (ClimaFruit) and by the Danish Council for Independent Research | Technology and Production Sciences (Project No. DFF-135-00182).

## Contributions by the Authors

L.A. and M.P. conceived the study and designed the experiment. U.B.A. and M.P. carried out the experiment. M.P. drafted the paper and all authors read, modified and approved the final manuscript.

## Conflict of Interest Statement

None declared.
